# Norman A. Johnson’s, Darwin’s Reach: 21st Century Applications of Evolutionary Biology

**DOI:** 10.1093/emph/eoad003

**Published:** 2023-03-03

**Authors:** Michael A Goldman

**Affiliations:** Department of Biology, San Francisco State University, San Francisco, CA 94132-1722, USA

There has always been some tension between the proponents of pure and applied science. Now, evolutionary biology joins virtually every other field of science in being dogged by practicality. Whether or not this has always been true (and I might argue that it has) is a matter for historical study, but today it is incontrovertible. In *Darwin’s Reach: 21st Century Applications of Evolutionary Biology*. Boca Raton, FL: CRC Press, 2022, ISBN 978-1-138-58739-7. $59.95 US, author Norman A. Johnson unabashedly celebrates this reality.

Darwin was always a bit impractical. He didn’t accept the common gentlemanly occupations of his day, such as theology or medicine. In time, however, Darwin’s thinking would revolutionize these and practically every other field of inquiry. Whether Darwin knew it or not, his work itself was rooted in practicality. The most direct way of observing natural selection in action came from his studies of variation under domestication, which rightly occupies the first chapter of *The Origin of Species*. If there was a time when evolutionary biology didn’t have an applied side, it might be in the time before Darwin, when incorrect notions of the inheritance of acquired characters threatened our ability to manipulate nature. The Lysenko affair makes this abundantly clear. Failure to accept the basics of genetics and natural selection has always been at our own peril. Yet one-third of Americans don’t accept the evidence for evolution. (One in five or so think COVID-19 is a hoax.) Education about genetics and evolution remains sparse, particularly misunderstood by many practitioners of medicine, despite the pervasive importance of these fields in our everyday lives.

Johnson gives much of the credit for evolutionary medicine to Randy Nesse, an accomplished leader and thinker in the field: [Preface xiii]. In the 1990s, George Williams and Randy Nesse, followed by others, developed Darwinian (evolutionary) medicine, applying the principles of evolutionary biology to the broader question ‘Why do we get sick?’ In the time since, evolutionary medicine is becoming a separate discipline, with its own various institutes, centers, and journals. I look back at least a few decades earlier to the work of Anthony Allison (1954) on sickle cell anemia, which connected a gene important in medical genetics to the process of natural selection, suggesting that the frequency of sickle cell anemia is higher in regions where *P. falciparum* malaria is common because of the protection afforded against the latter disease.

Fittingly, Johnson delayed the publication of his book in order to add a chapter about COVID-19, which was emerging as he wrote. It’s the union of two things a surprising number of Americans do not believe in, but it’s occupied the news cycles for more than 3 years. Viruses like COVID-19 provide a contemporary laboratory for rapid evolution under natural selection. At the same time as the viruses respond to natural selection, mutation, migration and genetic drift, the human hosts are themselves under selection to avoid the worst consequences of infection, either through environmental manipulation or the unforeseen inheritance of advantageous genetic variants. Johnson covers thoroughly the correlations with human genetics we knew at the time—the ABO blood groups, the secretor locus, and a region of chromosome 3, studied by Savante Paabo and Hugo Zeberg, that includes a Neanderthal variant conferring increased risk for poor COVID-19 outcome. Work after publication by Zeberg suggests that the same region may have been under positive selection by smallpox centuries earlier.

Perhaps the most important chapter, in view of the contemporary political tensions in America, is that on ‘Human genetic diversity and the non-existence of biological races’, which the author puts in the context of very recent and deep historical manifestations of racism in the USA. Johnson states categorically ‘… there is no good biological criterion to place humans into races, as we can for chimpanzees and some other animals. There is only one human race’. He cites work of Richard C. Lewontin, dating back to 1972, showing that only about 6% of human genetic variation can be explained on the basis of traditional ‘racial’ assignments, and more recent studies put that value at 3–5%. Dog breeds, on the other hand, explain more than 25% of genetic variation. An often-cited phenotypic characteristic of race, skin color, represents the same sort of response to selective pressures that we see in variants related to susceptibility to diseases like smallpox and sickle-cell anemia. The competing selective forces here are the need for vitamin D, which favors reduced pigmentation in low-UV environments, and the importance of avoiding folate degradation in high-UV environments (which would result lower fertility and in birth defects), favoring high melanin production in these regions. ‘We are much more alike than we are different’, Johnson concludes.

Johnson undertakes a wide variety of topics, many of which have not been mentioned in this review, including evolutionary medicine, pandemics like COVID-19 and HIV/AIDS, mosquito-borne diseases, personalized medicine, cancer, agriculture, biodiversity, and invasive species. He concludes with a few chapters on evolution and society, including genetic analysis in criminal investigations, warfare, and the non-existence of human biological races. While it is not encyclopedic, it is hard not to label the book comprehensive. The book is organized into chapters that can be read as independent episodes, with numbered footnotes at the end of each chapter referring to a larger bibliography at the end of the book, and it is well indexed. I found the book useful in preparing a few lectures for my general evolutionary biology course, but the coverage of elementary information is too sparse to give it potential as a textbook. However, I would not hesitate to suggest it as supplementary reading for interested undergraduates. It should provide useful insights for evolutionary biologists, geneticists and health professionals.

In universities, we put applied and non-applied fields in distinct colleges. Schools of law, medicine, dentistry and engineering are off by themselves. The ‘pure’ sciences may get to be with the humanities and arts, but the health-related sciences, agriculture, and education get colleges of their own. What then when we see that the esoteric field of evolutionary biology can be applied to some of the most crucial problems in healthcare, adaptation to climate change, sustainable production of energy and the cultivation of nutrient-rich foods in an environmentally responsible way? We talk about the interdisciplinary approaches needed. Now it’s time to do something about them, and the infusion of disciplined evolutionary thinking into a broad range of fields, only a few of them touched upon here, is a good place to start.

